# Supplementary material for: Identification of signalling pathways involved in gill regeneration in zebrafish
Source: J Exp Biol. 2024 Jan 26;227(2):jeb246290. doi: 10.1242/jeb.246290 (PMC10906665; doi:10.1242/jeb.246290)
Supplement: Supplementary information [file jexbio-227-246290-s1.pdf]

Table S1. Primer pairs used for mRNA quantification by RT-PCR.

Gene	Primer Sequence (5'-3')	Reference
fgf8a	F: GCCGTAGACTAATCCGGACC<br>R: TTGTTGGCCAGAACTTGCAC	Gebuijs et al., 2019
bmp2b	F: AGGCTGGAATGACTGGATTG<br>R: TTGCTGTTCACCGAGTTCAC	Nesan et al., 2012
bmp6	F: AACCGCAACCGCTCCAATAG<br>R: AACGCACCACCATGTTCCTG	Li and Ge, 2011
her6	F: TGAACTCGGGACACTTCGTG<br>R: GCACAGCTGCTTCTAGTGGA	Huang et al., 2019
jag1b	F: CGCTAAGTCATGCCACAA<br>R: TCCACATCCTTCTCACACT	Ma and Zhang, 2015
shha	F: AAGCCCACATTCATTGCTCT<br>R: CCTCTGTCCTCCGTCCTG	Sugimoto et al., 2017
ef1a	F: GGGCAAGGGCTCCTTCAA<br>R: CGCTCGGCCTTCAGTTTG	Robertson et al., 2014

F: forward primer, R: reverse primer.

Journal of Experimental Biology • Supplementary information

References

Gebuijs, I. G. E., Raterman, S. T., Metz, J. R., Swanenberg, L., Zethof, J., Van Den Bos, R., Carels, C. E. L., Wagener, F. A. D. T. G. and Von Den Hoff, J. W. (2019). *Fgf8a* mutation affects craniofacial development and skeletal gene expression in zebrafish larvae. *Biol. Open* 8, bio039834. doi:10.1242/bio.039834

Huang, V., Butler, A. A. and Lubin, F. D. (2019). Telencephalon transcriptome analysis of chronically stressed adult zebrafish. *Sci. Rep.* 9, 1379. doi:10.1038/s41598-018-37761-7

Li, C. W. and Ge, W. (2011). Spatiotemporal expression of bone morphogenetic protein family ligands and receptors in the zebrafish ovary: a potential paracrine-signaling mechanism for oocyte-follicle cell communication. *Biol. Reprod.* 85, 977-986. doi:10.1095/biolreprod.111.092239

Ma, W. R. and Zhang, J. (2015). Jag1b is essential for patterning inner ear sensory cristae by regulating anterior morphogenetic tissue separation and preventing posterior cell death. *Development* 142, 763-773. doi:10.1242/dev.113662

Nesan, D., Kamkar, M., Burrows, J., Scott, I., Marsden, M. and Vijayan, M. (2012). Glucocorticoid receptor signaling is essential for mesoderm formation and muscle development in zebrafish. *Endocrinology* 153, 1288-1300. doi:10.1210/en.2011-1559

Robertson, C. E., Wright, P. A., Köblitz, L. and Bernier, N. J. (2014). Hypoxia-inducible factor-1 mediates adaptive developmental plasticity of hypoxia tolerance in zebrafish, *Danio rerio*. *Proc. Biol. Sci* 281, 20140637. doi:10.1098/rspb.2014.0637

Sugimoto, K., Hui, S. P., Sheng, D. Z. and Kikuchi, K. (2017). Dissection of zebrafish *shha* function using site-specific targeting with a Cre-dependent genetic switch. *eLife* 6, e24635. doi:10.7554/eLife.24635
